# Estimating Heart Rate and Respiratory Rate from a Single Lead Electrocardiogram Using Ensemble Empirical Mode Decomposition and Spectral Data Fusion

**DOI:** 10.3390/s21041184

**Published:** 2021-02-08

**Authors:** Iau-Quen Chung, Jen-Te Yu, Wei-Chi Hu

**Affiliations:** 1 The Department of Biomedical Engineering, Chung Yuan Christian University, Taoyuan City 32023, Taiwan; g10002503@cycu.edu.tw; 2 The Department of Electrical Engineering, Chung Yuan Christian University, Taoyuan City 32023, Taiwan; yu@cycu.edu.tw

**Keywords:** electrocardiogram (ECG), photoplethysmogram (PPG), ensemble empirical mode decomposition (EEMD), intrinsic mode function (IMF), principal component analysis (PCA), ensemble empirical mode decomposition with principal component analysis (EEMD-PCA), ensemble empirical mode decomposition with spectral data fusion (EEMD-SDF), heart rate (HR), respiratory rate (RR), ECG derived respiration (EDR)

## Abstract

Cardiopulmonary monitoring is important and useful for diagnosing and managing multiple conditions, such as stress and sleep disorders. Wearable ambulatory systems can provide continuous, comfortable, and inexpensive means for monitoring; it always has been a research subject in recent years. Being simple and cost-effective, electrocardiogram-based commercial products can be found in the market that provides cardiac diagnostic information for assessment, including heart rate measurement and atrial fibrillation identification. Based on a data-driven and self-adaptive approach, this study aims to estimate heart rate and respiratory rate simultaneously from one lead electrocardiogram signal. In contrast to ensemble empirical mode decomposition with principle component analysis, performed in the time domain, our method uses spectral data fusion, together with intrinsic mode functions using ensemble empirical mode decomposition obtains a more accurate heart rate and respiratory rate. Equipped with a rule-based selection of defined frequency levels for respiratory rate (RR) estimation, the proposed method obtains (0.92, 1.32) beat per minute for the heart rate and (2.20, 2.92) breath per minute for the respiratory rate as their mean absolute error and root mean square error, respectively outperforming other existing methods.

## 1. Introduction

Heart rate and respiratory rate, two out of five vital signs, are usual monitoring for individual health status in the Internet of Things (IoT) era [1]. For single-lead ECG, one of these IoT-based wearable devices has shown many applications, such as the ECG-derived respiration (EDR) rate over past years, using different techniques on features, such as R-amplitude, QRS complex, RS slope, etc., which are sensitive to noise or artifacts during continuous ambulatory monitoring [2]. On the other hand, a single-lead ECG signal is one of the major candidates for promoting the opportunities of telemonitoring by measuring the heart rate, cardiac rhythm, and even detection of atrial fibrillation (AF) in chronic disease [3,4,5]. However, these devices still face some issues, such as power consumption, and a certain degree of inaccuracies, etc. As such, accuracy improvement and enhancement of data processing efficiency on the device still remain interesting and challenging research topics in this area.

Respiratory rate (RR) is not only a biomarker of monitoring deterioration in the intensive care unit, but also a significant vital sign of earlier diagnostic purpose in the general ward [6]. Most of these measurements of respiratory signals, such as impedance pneumography, are inconvenient and uncomfortable for patients and unsuitable for ambulatory monitoring [7]. In the case of pervasive and remote monitoring, ECG and PPG are two major candidates. PPG signal might be more advantageous than ECG signal, due to its simplicity, portability, and a small number of sensors. However, ECG signal has superior performance than PPG signal on monitoring heart activities, and PPG signal is only suitable for average or moving average measurements. A study also demonstrated that respiratory pulse rate variability could not precisely reflect respiratory heart rate variability in standing subjects and patients with low heart rate variation (HRV) [8,9]. Similarly, some researchers reported that PPG-based devices could not be applied as a precision screening tool to detect HRV [10]. ECG and PPG signals are primarily cardiopulmonary origin, with secondary respiratory modulations of much lower magnitudes, thus, heart rate (HR) extraction from these signals modulated by respiratory activity is more easily than RR. In general, techniques for extraction of a respiratory signal fall into two categories: Filter-based or feature-based [11]. Some of the filter-based techniques were employed on the elimination of frequency content outside of the range of plausible respiratory frequencies by wavelet transform and band-pass filtering [12,13]. The numbers of feature-based algorithms developed are greater than the ones of filter-based algorithms over past years, more used mixed method of both techniques to obtain more accurate breathing rate. An assessment of evaluating the previous algorithms to estimate respiratory rate from PPG and ECG signals reported that the performance of the algorithms using ECG are performed better than PPG [14]. The estimations of respiratory rate, regardless of the ECG or PPG signals, have their limitations. As to filter-based techniques, for example, digital filters of band pass filtering [15], the main problem of this technique was to realize the order of the filters and selection of the exact frequency band for filtering. Another filter-based wavelet approach suffered from the selection of mother wavelet, the level of decomposition, and performance reliability [16]. ECG has comprehensive features, such as R amplitude, and QRS complex, etc. [2]; these successful features depend on the precision of locating the position R peak and Q trough and S trough in enough resolution of time. Therefore, the feature-based approach is very noise sensitive, and the performance of the technique is highly affected by artifacts. If the amplitude of the respiratory signal is too small compared to the underlying noise, then the signal may not be distinguishable from the noise [17].

Empirical mode decomposition (EMD) is a self-adaptive and data-driven method proposed by Huang et al. [18], can be applied to study the non-linear and non-stationary properties of a time series. Earlier research reported that EMD is suitable for reconstruction of the respiratory waveform from ECG, can be a prominent alternative for the indirect monitoring of respiratory activity [19]. A report used the phase synchronization index to evaluate the cardiopulmonary coupling, which is present by the series of ECG-derived respiration and R peak to R peak interval, based on the EEMD decomposition from the ECG signal in analyzing cardiorespiratory coupling characterized by non-linear dynamics and non-stationarities [20]. Another report used EEMD to estimate the dominant frequency of the atrial activity signal, which is one of the most relevant features is characterizing atrial fibrillation, is reported by previous research [21]. However, a well-known limitation of the EMD method is caused by intermittent signals and noises, and it creates serious aliasing in time-frequency distribution and makes the physical meaning of individual intrinsic mode function (IMF) ambiguous. To overcome the mode-mixing problem of EMD, an improved EMD called as Ensemble EMD was introduced [22] to add white noise into the signal with different noise in each trial, more and more trials added to the ensemble, and decomposed the add-noised signal into IMFs, which followed the same processes as EMD. Both wavelet and EMD techniques are classified as methods of filter-based decomposition, and a comparison was present in a study [23].

Data fusion is an assisted step to improve the performance of EDR. It is often classified into three categories: The low level fusion (LLF), intermediate level fusion (ILF), and high level fusion (HLF). The LLF combines raw data sources to provide better information. The ILF combines features that come from heterogeneous or homogeneous raw data. The HLF combines decisions or confidence levels coming from several experts (hard and soft fusion) [24]. Principal component analysis (PCA) is a popular tool for dimension reduction, which is a data fusion as well. A pioneered study introduced PCA to develop EDR [25] reported that they investigated the morphological beat-to-beat variations and applied the PCA to the QRS complex, took the eigenvectors as the EDR signal. Later, another research [26] similarly used Kernel PCA to improve the non-linearities in the data. Recently, using PCA on the EDR, a report [27] illustrated the difference between its method and [25] is the former used feature-in feature-out data fusion, whereas the latter used data-in data-out data fusion; thus, first component of the PCA matrix which is composed of features represented the EDR. All these previous studies used PCA in the time domain. In addition, a study using data fusion in frequency from two different methods of R amplitude and RS amplitude using auto-regression estimation comes up with an improve results [28]. Similarly, using the nature of correlation technique to calculate the product of the spectra of three different feature-based methods of EDR, which is a type of spectral data fusion, it is reported that the spectral fusion method outperforms the individual methods considering all the metrics [29].

Our study aims to propose an approach that is based on ensemble empirical mode decomposition with spectral data fusion (EEMD-SDF) to estimate HR and RR simultaneously from a single-lead ECG signal. It is inspired by the method of ensemble empirical mode decomposition with principal component analysis (EEMD-PCA) for simultaneous estimation of HR and RR from PPG signals [30]. EEMD is able to break down the ECG without the preselected basis function and removes the mode mixing problem. EEMD-PCA is a method in the time domain, EEMD-SDF is an approach in the frequency domain, and both of them yield similar estimations of HRs, but comparably RRs. In addition, EEMD-SDF saves more computing costs than EEMD-PCA, which is illustrated in later sections.

This paper is organized as follows. Section 2 introduces the data sets, the processes of the proposed algorithm, and evaluation performance metrics. In Section 3, the results of the proposed method using data sets are reported. The discussion and conclusion are illustrated in Section 4 and Section 5, respectively.

## 2. Materials and Proposed Method

The test data from the Vortal dataset [14] and the proposed processing method, including the associated processing stages, are detailed below.

### 2.1. Dataset

This study uses data from the Vortal dataset [14], which contains electrocardiogram (ECG), photoplethysmogram (PPG), impedance pneumography (imp), and reference oral-nasal pressure (paw) signals acquired from young subjects (aged 18–39) and elderly subjects (aged 70+). The ECG data in this study were collected under a 500 Hz sampling rate via a clinical monitor. All of the subjects are in good health condition. Data were acquired for approximately 10 min while lying supine at rest. In this study, each data segment extracted from a subject has 60 s in length. Six consecutive segments for a subject, each segment overlapped with the previous one by 6 s. As such, there are 342 segments for studies.

### 2.2. The Proposed Method

**Figure 1:**
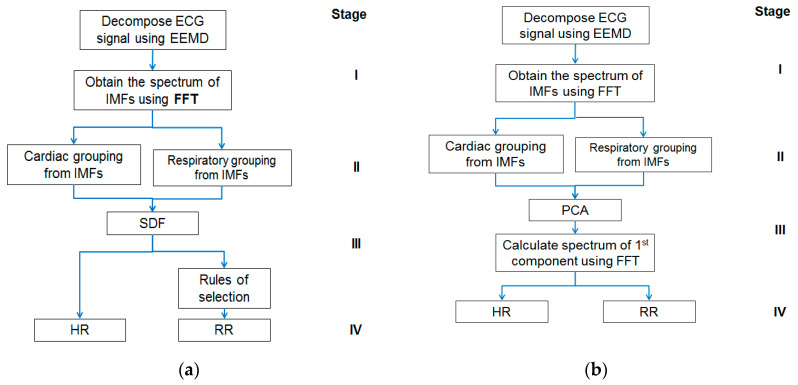
(**a**) The block diagram of the ensemble empirical mode decomposition with spectral data fusion (EEMD-SDF) method; (**b**) the block diagram of the ensemble empirical mode decomposition with principal component analysis (EEMD-PCA) method. FFT, Fast Fourier Transform; RR, respiratory rate; HR, heart rate; IMF, intrinsic mode function.

The approach has two appealing features. Firstly, the ECG signal decomposition is self-adaptive and data-driven; hence, a priori functions for data processing are not needed. Secondly, the HR and RR estimations are obtained through simple calculations performed in the frequency domain followed by rule-based selections leading to less computational complexity and demand. However, this study may have its limitation too on low RR. The minimum RR, which is 5 BPM in this study, determines the number of IMFs for the respiratory group and may require selection rule change. Further investigation, therefore, is needed in the future to verify if the proposed approach is still applicable to the detection of very low RR, such as that in the case of obstructive sleep apnea (OSA). Given below is Figure 1 showing block diagrams of the EEMD-SDF method and the EEMD-PCA method, respectively.

The proposed method comprises four stages, as shown in Figure 1a: (I) Using EEMD to decompose ECG signal, followed by Fast Fourier Transform (FFT), (II) grouping IMFs by the frequency range of HR and RR, (III) using SDF to calculate superposition of spectra of the grouped IMFs to estimate HR, and followed by selection rules to get RR (IV) estimating HR and RR. Further details of the above four stages of the EEMD-SDF method are provided below.

#### 2.2.1. Using EEMD to Decompose the ECG Signal

EEMD decomposes a signal by the following steps:

Add a white noise series n(t) to the signal s(t) and let s1(t) = s(t) + n(t).

Set s1(t) as signal x(t) and follow step 3 through step 9 as in EMD

Find all local maxima and minima of x(t).

Generate the upper and lower envelopes from those maxima and minima of step 3 by cubic spline interpolation.

Calculate the mean function m(t) of the upper and lower envelopes.

Calculate the difference d(t) = x(t) − m(t).

If d(t) becomes a zero-mean signal, then stop as d(t) is IMF1, denote which as c_1_(t); otherwise, go to step 3 and replace x(t) by d(t).

Find the residual signal r(t) = x(t) − c_1_(t).

Repeat steps 3 through 8 to obtain IMF2, denote which as c_2_(t). Repeat steps 3–8 for n times to obtain c_n_(t). Stop the procedure when the final residual signal r(t) becomes a monotonic function.

A residual signal r(t) and a collection of IMFs c_1_(t) to c_n_(t) are obtained at the end of the procedure. The original signal can now be represented by

(1) $$x\left(t\right)=\overset{n}{\underset{i=1}{\sum }}c_{i}\left(t\right)+r\left(t\right)$$

In fact, r(t) can be regarded as c_n+1_(t).

Repeat Steps 1 through 9 till the trial number with different white noise series having the same added power. A new IMF C_ij_(t) is obtained where the subscripts “i” refers to the iteration number, and “j” stands for the scale, respectively.

Estimate the mean (ensemble) of the final IMF as the desired output

(2) $${\mathrm{EEMD}}_{c_{\mathrm{ij}}\left(t\right)}=\overset{j=k}{\underset{j}{\sum }}c_{\mathrm{ij}}\left(t\right)$$

where k denotes the trial number.

**Figure 2:**
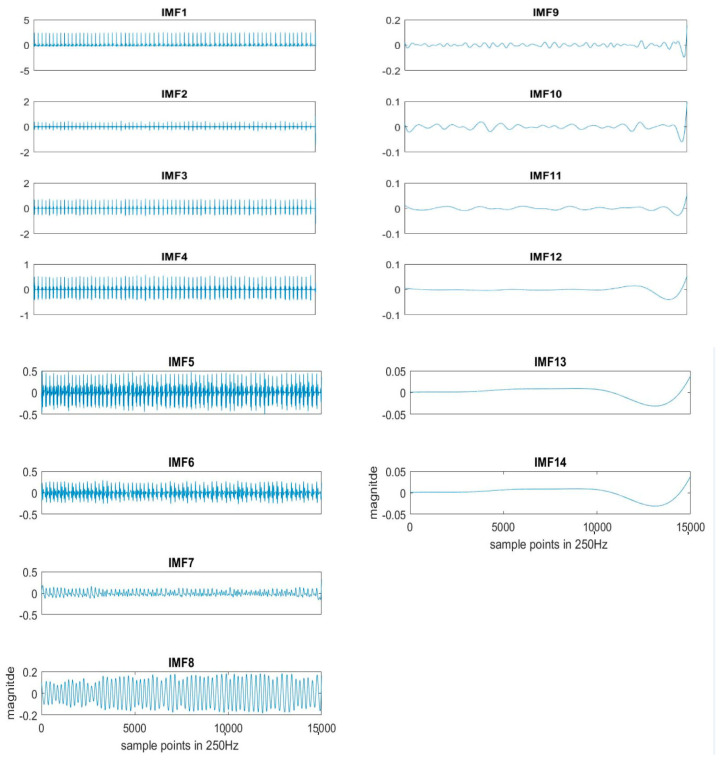
An example of IMFs decomposed by EEMD sifting process from the ECG signal.

Here k = 20 is used for the experiment. Each segment of ECG is decomposed to obtain the IMFs, as shown in Figure 2. The original ECG signal is IMF1.

#### 2.2.2. Grouping IMFs for HR and RR

**Figure 3:**
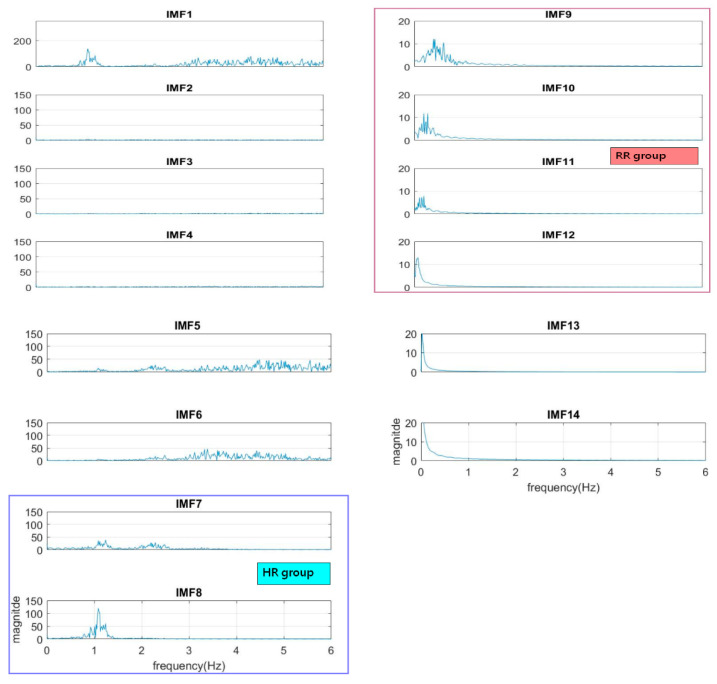
The spectra of IMFs (same one as in Figure 2).

The normal range of HR for adults is between 60–100 beat per minute (BPM). In general, a lower heart rate at rest implies more efficient heart function and better cardiovascular fitness. The rate can be affected by factors like stress, anxiety, hormones, medication, and physical activities. The normal respiration rate for an adult at rest is 12 to 20 BPM (breath per minute). A respiratory rate under 12 or over 25 in rest is considered to be abnormal. For 2 to 18 years old children and young adults’ normal range of RR and HR typically runs from 8 to 45 breaths/min and from 45 to 145 beats/min, respectively [31]. As such, it is reasonable to set the frequency as 0.08–0.75 Hz, i.e., 5–45 BPM for RR and 0.75–2.5 Hz, i.e., 45–150 BPM for HR. Following the frequency ranges of HR and RR, the IMFs of RR and HR are selected to have maximum spectrum power within these ranges, as shown in Figure 3. The rules of selection will be included in the EEMD-SDF processing in the sequel when EEMD-SDF and EEMD-PCA are compared in RR estimation.

To reduce computational cost while preserving the features of ECG, the data is down-sampled, with frequency dropping from 500 Hz to 250 Hz. The segment of ECG signal decomposed by EEMD with this lower sampling rate has 14 IMFs. It is found that IMF7 and IMF8 are always related to the cardiac group, as revealed by Figure 3. In addition, power is used to find the key IMF in the HR within the cardiac group, the criterion being that the one with larger power is the dominant IMF. Similarly, IMF9–IMF12 within the respiratory frequency range is selected to be of the RR group. Once the IMFs of HR and RR is defined, there is no need to calculate the spectrum of IMFs other than IMF7–IMF12.

#### 2.2.3. IMFs Mapping to Frequency Levels

For a normal adult, the range of RR is 12–20 BPM, which is further categorized in three frequency levels, as shown in Table 1.

Shown in Table 2 is the frequency distribution of IMF9–IMF12 wherein IMF9–IMF11 can be mapped to H, M, and L, respectively, according to Table 1.

According to the respiratory frequency level, it is hypothesized that the estimated RR pertains to one of IMF9–IMF11 from Table 2. Another hypothesis is based on reasonable physical meaning: The dominant IMF in the cardiac group must be the one having larger power contributing most to the HR. It is very rare that the dominant IMF for HR and the IMF representing RR happen to be two adjacent IMFs. As can be easily seen from Table 2 that the frequency of the IMF is almost doubled compared to the next IMF. For example, if the HR is 68 BPM from the dominant IMF7, it is highly unlikely that RR is 34 BPM as being represented by IMF8. We may, therefore, assume that if the dominant IMF of HR is IMF7 (or IMF8), then the dominant IMF of RR is IMF9 (or IMF10).

#### 2.2.4. Spectral Data Fusion of IMFs for HR and RR

SDF is about the fusion of spectrum. The HR and RR from the ECG signal in this study are estimated by superposition of the spectra of related IMFs. That is, we calculate the frequency having the highest power (FHHP) together with SDF from the cardiac group to estimate the HR. Likewise; the SDF is also employed on the IMFs pertaining to the RR group. We calculate the superposition of spectra of IMF9–IMF10 as RR_1, IMF10–IMF11 as RR_2, and IMF9–IMF11 as RR_3, respectively, to find the FHHP. RR_1, RR_2, and RR_3 plus the dominant IMF of RR in terms of the frequency levels (FLs) given in Table 1, are composed of variables of the rules of selection for the estimated RR. The processing of EEMD-SDF without the selection rule on IMF9–IMF11 in the respiratory group would be termed EEMD-RR_3.

#### 2.2.5. The Rules of Selection for RR Estimations

**Figure 4:**
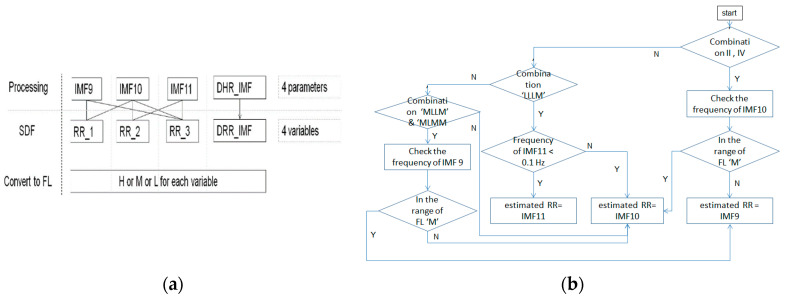
(**a**) The processing of SDF; (**b**) the flow of checking IMF10.

The basics are the prominent respiratory activity would render the related IMFs, which are the components of the four variables we defined. The steps of the rule-based selection of IMF as estimated RR from IMF9–IMF11 are listed as follows:

1. The frequencies of RR_1, RR_2, and RR_3 are the same; if the frequency appears in IMF9–IMF11, then the frequency is selected as estimated RR.
2. If the combinations of FLs are composed at least of two ‘H’ FLs and one from the DRR_IMF, then the IMF9 is selected as the estimated RR, as showing in Table 3, combination I.
3. If combinations II of FLs are not obvious to identify whether IMF9 or IMF10 is selected as the estimated RR, then check the frequency of IMF10 according to the rule, as shown in Figure 4, to select the IMF.
4. If the combinations of FLs are composed at least of three ‘M’ FLs, then the IMF10 is selected the estimated RR, as shown in Table 3, combination III.
5. If the combinations of FLs are composed at least of two ‘L’ FLs among RR_1-RR_3, and DRR_IMF is ‘M’, then the IMF10 is selected as the estimated RR, as shown in Table 3, combination IV
6. If the combinations of FLs are not regular, which means the combinations are odd and not meaningful, as shown in Table 3, combination V, then check the frequency of IMF10 in Figure 4, which is to be selected as estimated RR.
7. If the combination of FL is ‘LLLM’, then it needs to check IMF11 further. If the FL of IMF11 is ‘L’, then the IMF11 is selected as the estimated RR, as shown in Figure 4b.
8. If the combination of FL is ‘MLLM’ or ‘MLMM’, then it needs to check IMF9 further. If the FL of IMF9 is ‘M’, then the IMF9 is selected as the estimated RR, as shown in Figure 4b.
9. For outliers handling, if the outliner is over 5.5 BPM above the mean value of 3 previous values, then the previous one is selected to replace the currently estimated frequency (outlier).

#### 2.2.6. The Optimal RR

As a hypothesis aforementioned, the estimated RR comes from one of the IMF9–IMF11. The estimation of optimal RR is to manually find which the frequency of IMF in the respiratory group is closest to the reference RR. For example, the frequencies of IMF9–IMF11 are 0.3815, 0.2289, 0.1526 Hz, respectively. Since the reference RR is 21 BPM, the optimal RR for this segment, therefore, is 0.3815 Hz, which is 23.25 BPM.

#### 2.2.7. EEMD-PCA Method

The approach was proposed in a previous study [30]. It extracts the surrogate signals of HR and RR from PPG signal using EEMD decomposition followed by grouping related IMFs within the frequency bands of cardiac and respiratory activities, respectively. The surrogate signals are processed by PCA to obtain the first component, on which FFT then is employed to estimate HR and RR. As can be seen from stage I and II of Figure 1, both EEMD-PCA and EEMD-SDF yield the same results. Their main difference lies in stage III, in that the computation of the former is performed in the time domain, whereas the latter is in the frequency domain, which is computationally less demanding, hence more appealing.

#### 2.2.8. Estimation of HR and RR

Once the estimated frequencies of HR and RR are obtained, they are converted into BPM according to Equations (3) and (4) as

HR = est. HR ∗ 60 (beats/min)(3)

RR = est. RR ∗ 60 (breaths/min)(4)

In our study, the reference RRs are from the signals of oronasal and impedance (IP) as in the Vortal dataset. If inconsistencies are found, we examine their waveforms to count the RR manually and find the rights RRs. Likewise, the reference HRs are calculated manually from the ECG signals.

### 2.3. Performance Measures

Five measures are defined in this paper for performance assessment. Mean Absolute Error (MAE) is a simple measure to evaluate performance. By definition, the mean absolute error is the mean of the absolute value of the difference between the estimated value and the actual one. A shortcoming of MAE is that the relative magnitude of the error is not visible. The relative MAE (rMAE) of Equation (6) or mean absolute percentage error (MAPE) allows us to compare the errors of two series on different scales, which are defined as

(5) $$\mathrm{MAE}=\frac{1}{N}\overset{N}{\underset{1}{\sum }}|x_{i}-x_{r}|$$

(6) $$\mathrm{rMAE}=\frac{1}{N}\overset{N}{\underset{1}{\sum }}|x_{i}-x_{r}|/x_{r}$$

The estimated HR or RR is denoted as x, and the referenced HR or RR as x_r_ where N refers to the number of data points. To assess the estimation, the root mean square error (RMSE) of Equation (7) as a metric is computed and expressed as

(7) $$\mathrm{RMSE}=\sqrt{\frac{1}{N}\overset{N}{\underset{1}{\sum }}{\left(x_{i}-x_{r}\right)}^{2}}$$

We use MAE, rMAE, and RMSE to extract the variations in the errors. The RMSE is larger or equal to the MAE. In addition, Box-Whiskers Plot with five sample statistics, including the minimum, the lower quartile, the median, the upper quartile, and the maximum, are presented. The box is a rectangle that encloses the middle half of the sample and ends at the quartile. The length of the box represents the interquartile range (IQR) of the sample.

The difference between the proposed algorithm and the reference is assessed using the Bland–Altman scheme. This method calculates the mean difference between two methods of measurement, and 95% limits of agreement (LoA) as the mean difference (2 SD), which is 1.96 SD more precision. The presentation of the 95% limits of agreement is for visual judgment of how well two methods of measurement agree. A smaller range between these two limits implies better agreement.

## 3. Results

The MAE, rMAE, and RMSE of the estimated RR and HR using EEMD-SDF and EEMD-PCA from the ECG signal are shown in Table 4 and Table 5, respectively. To eliminate inconsistency during the experiment, some of the irregular segments, for example, the FL ‘LLLM’ having the frequency of FL ‘H’ and ‘MMMM’ having the frequency of FL ‘L’, from 57 subjects are removed for 25 data segments; hence, the remaining 317 segments are processed.

**Figure 5:**
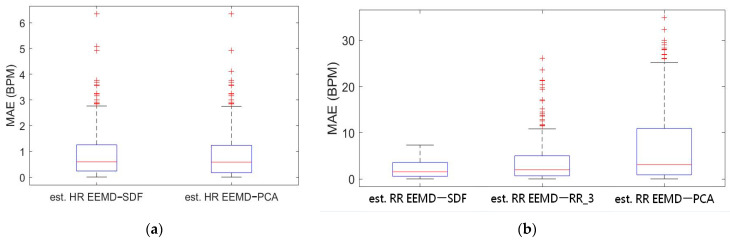
(**a**) Boxplot of MAE bias of the estimated HR using EEMD-SDF and EEMD-PCA; (**b**) boxplot of MAE of the estimated RR using EEMD with SDF, RR_3, and PCA.

Box-whiskers plots of the MAE of estimated HR and RR using the EEMD-SDF and the EEMD-PCA against the reference signal are in Figure 5a,b, respectively. From Figure 5a, it is found that the estimated HRs using the EEMD-SDF and the EEMD-PCA are almost the same. One can see from Figure 5b that the estimated RR using the EEMD-SDF outperforms the EEMD-RR_3 and EEMD-PCA.

**Figure 6:**
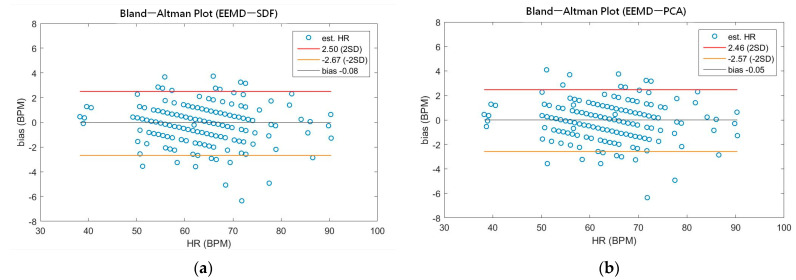
(**a**) The Bland-Altman plot of the estimated HR using EEMD-SDF; (**b**) the Bland-Altman plot of the estimated HR using EEMD-PCA.

**Figure 7:**
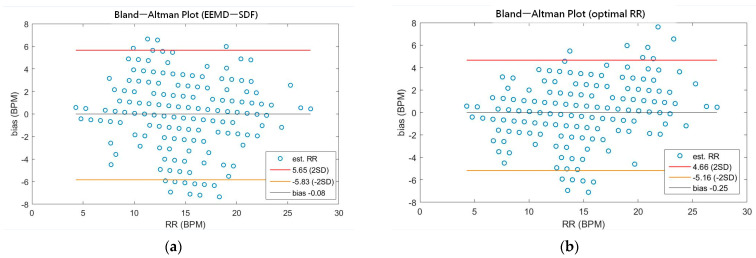
(**a**) The Bland-Altman plot of the estimated RR using EEMD-SDF; (**b**) the Bland-Altman plot of the optimal RR.

**Figure 8:**
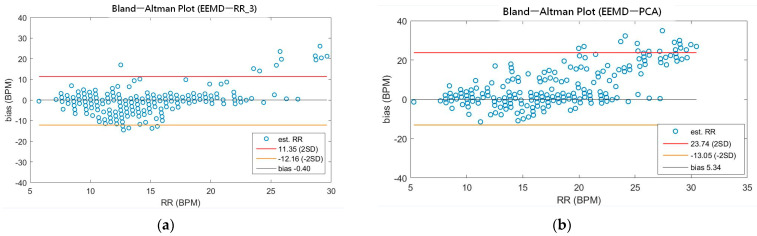
(**a**) The Bland-Altman plot of the estimated RR using EEMD-RR_3; (**b**) the Bland-Altman plot of the estimated RR using EEMD-PCA.

As shown in Table 4, the MAEs, rMAE, and RMSEs of HR are (0.96, 1.37%, 1.34) and (0.94, 1.38%, 1.33) using EEMD-SDF and EEMD-PCA. These results are almost the same in the estimation of HR, which also can be seen from Figure 5a. The estimated RR using the EEMD-SDF, however, outperforms the EMMD-PCA, as revealed by Table 4. For further investigation, we apply the Bland–Altman plot to check the agreement level of these two methods in the estimation of HR and RR whose results are shown in Figure 6 and Figure 7, respectively. From Figure 6, one can observe that the estimated HRs using the EEMD-SDF and the EEMD-PCA are very close, only two out of the 201 segments exhibit different HR estimations. The optimal RR in Table 5 is estimated manually with 87% accuracy as of rMAE. In addition, the estimated RR using the EEMD-RR_3, the method of EEMD-SDF without the rule of selection when to apply the estimation of RR, even outperforms the method of EEMD-PCA, as shown in Figure 8. The RR estimation, based on the EEMD-SDF method, is close to be optimal in this paper, as shown in Figure 7. The EEMD-SDF, on the other hand, reaches 83% of accuracy, according to Table 5.

## 4. Discussion

**Figure 9:**
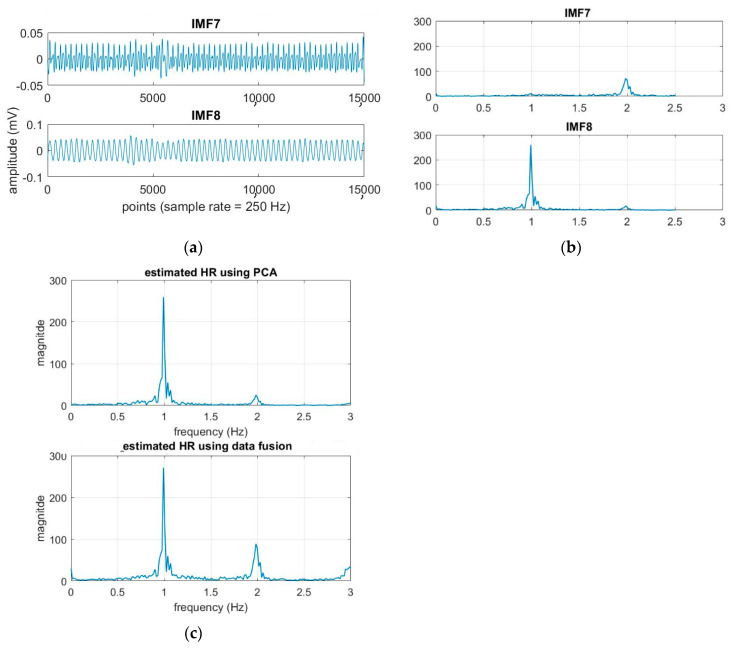
(**a**) The time series of the cardiac group; (**b**) the spectra of the time series; (**c**) the estimated HR using EEMD-PCA and EEMD-SDF.

From Table 4 and Figure 5a, it is found that both EEMD-SDF and EEMD-PCA yield the same estimated HR. The reason is that the waveforms of IMF7 and IMF8 are sinusoidal-like; hence, the estimated frequency from the largest variation of the time series of IMF7–IMF8 by PCA followed by FFT is the same as that of SDF, as shown in Figure 9. However, this finding is not present in the RR estimation, due to the nature of the time series IMF9–IMF11.

As can be seen from Figure 1, the main computational costs are different. In comparison with EEMD-PCA that applies FFT two times and matrix manipulation of PCA one time, EEMD-SDF only employs FFT one time plus a simple addition. Furthermore, PCA is computationally extensive, based on time complexity analysis, PCA processes composed of sequential matrix calculations, including covariance matrix and eigenvalue decomposition. Hence, the time complexity order of PCA is O(n^3^) (n is the number of basic operations), the more samples increase, the more computations need. However, the number of times of addition of spectra of IMFs and if-then-else operations of the rule selection employed on SDF would not change when samples increase, thus the time complexity of SDF is O(1) (constant computations). As such, EEMD-SDF, compared to EEMD-PCA, is less computationally demanding.

**Figure 10:**
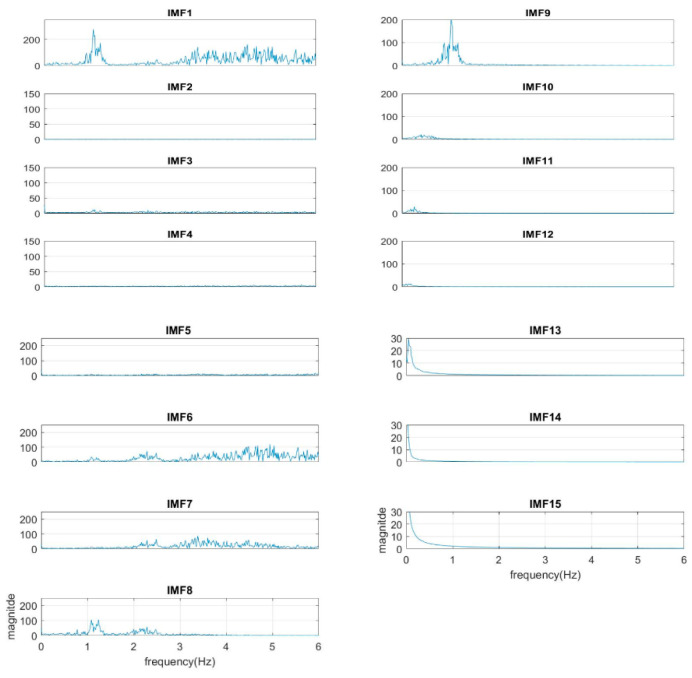
The spectra of IMFs with a sample rate of 500 Hz (same as the sample in Figure 3).

In our study, the sampling rate of the ECG signal is 250 Hz. We find from experiments that the number of IMFs will depend on the sample rate of the segment in fixed-length—the number of the IMFs will increase under a higher sample rate. For example, the number of IMFs is 15 under the sample rate of 500 Hz, but is 14 under 250 Hz. However, increasing the sample rate does not increase the resolution within cardiopulmonary bandwidth, as shown in Figure 10. (The segment is the same as that in Figure 3, but under a sample rate 500 Hz.) Apparently, the cardiac group shifts one IMF up from IMF7–IMF8 to IMF8–IMF9. The shifting goes to the respiratory group as well.

**Figure 11:**
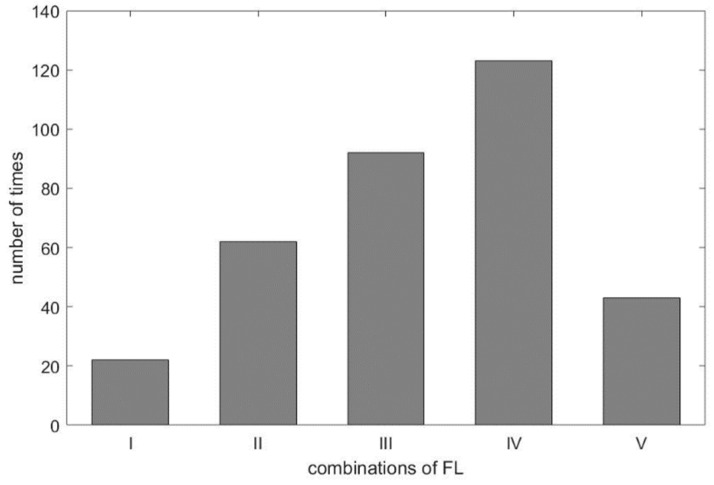
The distribution of frequency level combinations.

As shown in Table 3, we found the selected IMF9 and IMF10 to be the RR, due to apparent dominant frequency levels ‘H’ and ’M’ in the combinations I and III, respectively. For combination IV, two ‘L’ frequency levels among RR_1-RR_3 and one ‘M’ frequency level from DRR_IMF implies that IMF10 or IMF11, being mapped to ‘M’ and ‘L’, respectively, dominates the frequency of the respiratory activity. Interestingly, we found in the experiment the frequency of ‘L’ went to ‘M’, even in the combination of ‘LLLM’, hence IMF10 is selected as the estimated RR for combination IV. According to Table 1, the frequency of ‘M’ is from 12 BPM to 20 BPM. Some cases having frequencies close to the boundary values, such as 18, 19, or 21 BPM, lead to the so-called ‘balanced combination’, such as combination II, that has no dominant frequency level. It is also observed that the frequency of IMF10 in the experiment was below the range of ‘M’, which should go one level up to meet the classification, as shown in Figure 4. This rule applies to combination V as well. For visualization purpose, the distribution of combinations is plotted and shown in Figure 11. As expected, the ‘balanced combination II’ and ‘odd combination V’ occupy only a small proportion of all segments.

One can see from Figure 5a,b that the results from EEMD-SDF and EEMD-PCA are accurate, hence reliable in estimating HR. Some previous studies of EDR from signal lead ECG using MAE and rMAE metrics for evaluation instead of correlation and coherence statistics are considered and compared to our approach. A study [15] of respiratory activity derived from a single-lead portable ECG monitor with ten controlled 1-h recordings covering daily activities, such as lying, sitting, standing, walking, jogging, running, and stair climbing. The comparison is shown in Table 6. Another previous study [32] using the Fantasia dataset obtained EDR wherein the ECG and the respiratory recordings in rest condition. All signals are digitized by a sampling frequency of 250 Hz. Three features, labeled as M_MOM_, M_Slope_, and M_RSA_ in that study, are extracted then followed by the central moment technique to estimate RR. The mean values of MAE and rMAE for different segments and overlapped lengths are shown in Table 6 presented in a top-down order. Our approach outperforms the results of the study [15], and comparably good as ones of the report [32], as shown in Table 6, however, the later without recovered condition which may lead to larger variation in accuracy, as can be seen from results [15].

A recent assessment of the performance of the EDR algorithms (totally 314 algorithms, with 44 for ECG and 270 for both ECG and PPG) [17] reported that the best result has 95% LoAs of −4.7 to 4.7 bpm and a bias of 0.0 bpm on ECG. Impedance pneumography (IP), the clinical standard for continuous respiratory rate measurement in spontaneously breathing patients, has 95% LoAs of −5.8 to 5.4 bpm and a bias of −0.2 bpm. As revealed by Table 7, our approach has 95% LoAs of −5.83 to 5.65 bpm and a bias of −0.08 bpm, being very close to the IP. According to the assessment report [17], only four algorithms among all perform better than IP. To validate our approach against other methods using ECG signals, we summarize comparisons between the proposed method and other state-of-the-art methods in Table 7.

According to Reference [17] and Table 7, the algorithms ranked top 10 are feature-based that extract features from the data, such as the amplitudes of peaks and troughs, mean of these amplitudes, and the intervals of proceeding peaks. As mentioned above, these approaches suffer from noise and/or artifacts that could easily contaminate the signals [2,30] to make wrong estimations. It is observed from Table 7 that algorithms ranked top 4 (A–D) removed signals of bad quality to keep the accuracy up to a certain level, which can be seen from the values of the proportion of estimated data. Furthermore, algorithms (A–I) use auto-regression (AR), all-pole modeling (AR), or spectral analysis to find the pole with a maximum magnitude as a respiratory point. The disadvantage of these methods is that the order of the AR model must be chosen prior to estimation. In contrast, our approach using EEMD is data-driven without any need to preselect parameters and basis functions.

For optimal RR estimation, it is the first time, to our best knowledge, to find that an IMF within the respiratory frequency range 0.08–0.75 Hz can represent the respiratory activity. In addition, the proposed EEMD-SDF with selection rules and different combinations of defined FLs is a highly efficient method to identify the proper IMF and obtain the RR.

In clinical settings, monitoring now mostly uses impedance of IP (impedance pneumography) to measure respiratory signals. In breathing, the movement of the chest leads to the position change of the ECG electrodes on the skin resulting in a variation in impedance, which can be used to estimate the respiratory rate. Measuring the ECG impedance change for respiratory rate estimation requires some specialized hardware, however our scheme uses the ECG signal directly to derive respiratory rate without requiring any additional electronics. From a cost-effectiveness perspective, the proposed approach is more advantageous.

Applications of a single-lead ECG signal are popular. Currently, extractions of heart rate (HR) and respiratory rate (RR) from those cost-effective and wearable devices or patches are available in the market [33]. Besides, it is not only for monitoring primary health conditions, but also to assist diagnosis of cardiorespiratory diseases, including pneumonia, pulmonary embolism [34], and cardiac diseases [3]. Our approach provides a data-driven and self-adaptive way for simultaneous HR and RR estimation. As revealed by the experimental results, our scheme obtains better results compared to other methods according to the conclusion from previous studies. As such, the proposed may have the potential to be commercialized in the future.

## 5. Conclusions

In this study, we presented a spectral data fusion-based approach to estimate the heart rate and respiratory rate simultaneously from a single-lead ECG signal using EEMD techniques with a rule-based selection on RR estimation. Compared to other current methods, the estimated HR and RR are more accurate. Especially, our RR estimation has 95% LoAs of −5.83 to 5.65 bpm and a bias of −0.08 bpm, which is very close to the accuracy level of IP in a clinical setting. It is also found that the optimal RR, being obtained manually through the selection of IMFs decomposed by EEMD within a respiratory frequency range, can be used as a benchmark to evaluate other methods in choosing the proper IMF as the estimated RR. Furthermore, using the combinations of frequency levels of the defined variables to select an IMF as the estimated RR, the proposed approach can serve as a classifier, if the machine learning scheme is to be utilized.

## Figures and Tables

**Table 1:** Frequency level versus frequency range.

Frequency Level	Range (BPM)	Range (Hz)	RR Conditions
H	H > 20 BPM	H > 0.3333	greater than normal
M	12 < M ≤ 20	0.2 < M ≤ 0.3333	normal range of RR for adult
L	L ≤ 12 BPM	L < 0.2	less than normal

**Table 2:** The frequency distribution of IMF9-IMF11.

Item	IMF9 (BPM)	IMF10 (BPM)	IMF11 (BPM)	IMF12 (BPM)
median	23.80	12.81	7.32	4.57
maximum	44.86	27.46	13.73	7.32
minimum	14.64	8.23	4.57	4.57
frequency level	H	M	L	excluded

**Table 3:** The selection rule using combinations of frequency level.

	I	II	III	IV	V
	HLLH	HLHM	MLMM	MLLM	MLLH
FL	HLHH	HMHM	MHMM	LLLM	HLLM
combinations	HMHH	MLMH	MMMM	LMLM	LLLH
	HHHH	HLMM	MMMH	LHLM	
	MLHH HHHM		HMMM MMLM		
Selected IMF	IMF9	IMF9 or IMF10	IMF10	IMF10	IMF9 or IMF10

**Table 4:** The estimated HR using EEMD-SDF and EEMD-PCA.

	EEMD-SDF (BPM)	EEMD-PCA (BPM)
MAE	0.92	0.91
rMAE	1.46%	1.43%
RMSE	1.32	1.28
bias	−0.02	−0.07
LoA(±2 SD)	(−2.67, 2.50)	(−2.57, 2.46)

**Table 5:** The estimated RR using different methods.

	EEMD-SDF (BPM)	EEMD-RR_3 (BPM)	EEMD-PCA (BPM)	Optimal RR (BPM)
MAE	2.20	3.81	7.03	1.82
rMAE	17.02%	27.19%	55.77%	12.78%
RMSE	2.92	6.00	10.78	2.52
bias	−0.08	−0.46	5.34	−0.25
LoA(±2 SD)	(−5.83, 5.65)	(−12.16, 11.35)	(−13.05, 23.74)	(−5.17, 4.65)

**Table 6:** The comparison of RR using EEMD-SDF and other methods in MAE and rMAE.

Method	Age	Situation	MAE	rMAE (%)	Reference
EEMD-SDF	Healthy young and elderly mixed	rest and recovered mixed	2.2	17.0	The proposed approach
Band pass filter plus RSA	healthy young to middle aged	lying	2.0	18.0	[15]
		Recoverd1	3.1	16.0	
		Recoverd2	4.4	20.0	
Features extraction and central moment	young	rest and watch movie	2.0	12.2	[32]
			2.7	16.9	
			2.1	11.8	
	elderly		1.3	7.0	
			2.2	13.1	
			3.2	18.1	

**Table 7:** The comparison of using EEMD-SDF and other methods in limits of agreement (LoA).

Algorithm	Over All Rank	2SD (BPM)	Bias (BPM)	95% LoA	Proportion of Estimated Data (%)
The proposed approach	7	5.7	−0.08	−5.83 to 5.65	92.3
A	1	4.7	0.0	−4.7 to 4.7	73.8
B	2	5.2	1.4	−3.8 to 6.4	72.3
C	3	5.2	2.0	−3.3 to 7.2	75.4
D	4	5.3	1.4.	−3.8 to 6.7	72.5
clinical monitor	5	5.4	−0.2	−5.8 to 5.2	100
F	6	5.6	−0.2	−5.8 to 5.4	100
G	8	5.7	−0.2	−5.9 to 5.4	100
H	9	5.7	−0.2	−6.0 to 5.5	100
I	10	5.7	0.5	−5.2 to 6.3	100

## Data Availability

The data acquired is with consent of Peter H. Charlton and his institution by signing the agreement via the form ‘RESEARCH DATA ACCESS AGREEMENT FOR RESTRICTED DATA’ in July 2019. The relevant information of how to acquire dataset can be found https://peterhcharlton.github.io/RRest/vortal_dataset.html (accessed on 8 February 2021).
